# Hydrogels for the treatment of rheumatoid arthritis

**DOI:** 10.3389/fbioe.2022.1014543

**Published:** 2022-10-12

**Authors:** Jiafeng Yi, Yubo Liu, Hongbin Xie, Haoming An, Chao Li, Xing Wang, Wei Chai

**Affiliations:** ^1^ Senior Department of Orthopedics, Fourth Medical Center of People’s Liberation Army General Hospital, Beijing, China; ^2^ School of Medicine, Nankai University, Tianjin, China; ^3^ National Clinical Research Center for Orthopaedics, Sports Medicine and Rehabilitation, Beijing, China; ^4^ Beijing National Laboratory for Molecular Sciences, Institute of Chemistry, Chinese Academy of Sciences, Beijing, China; ^5^ University of Chinese Academy of Sciences, Beijing, China

**Keywords:** rheumatoid arthritis, hydrogel, drug delivery, transdermal delivery, biomaterials, tissue engineering

## Abstract

Rheumatoid Arthritis is a universal disease that severely affects the normal function of human joints and the quality of life. Millions of people around the world are diagnosed with rheumatoid arthritis every year, carrying a substantial burden for both the individual and society. Hydrogel is a polymer material with good mechanical properties and biocompatibility, which shows great potential in the treatment of rheumatoid arthritis. With the progress of tissue engineering and biomedical material technology in recent years, more and more studies focus on the application of hydrogels in rheumatoid arthritis. We reviewed the progress of hydrogels applied in rheumatoid arthritis in recent years. Also, the needed comprehensive performance and current applications of therapeutic hydrogels based on the complex pathophysiological characteristics of rheumatoid arthritis are also concluded. Additionally, we proposed the challenges and difficulties in the application of hydrogels in rheumatoid arthritis and put forward some prospects for the future research.

## Graphical Abstract

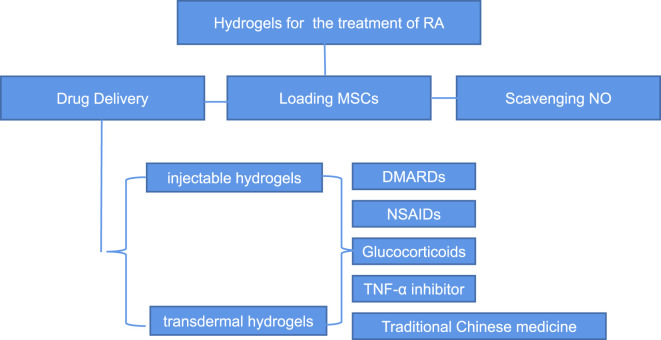

## 1 Introduction

Rheumatoid arthritis is an autoimmune disease that is characterized by persistent synovitis, systemic inflammation and autoantibodies (such as citrullinated peptide and rheumatoid factor) (David et al., 2010). The incidence rate of rheumatoid arthritis is 0.5%–1% and it decreases significantly from north to South (in the northern hemisphere). The urban areas have a higher incidence over rural areas (Josef et al., 2016).

The goal of diverse treatment strategies for rheumatoid arthritis is to relieve patients’ pain and inflammation, protect the normal joint function of patients, and prevent further progression of rheumatoid arthritis and joint damage (Matteson, 2000). Currently one of the mainly treatments is drug therapy including but not limited to the non-steroidal anti-inflammatory drugs (NSAIDs), disease modifying antirheumatic drugs (DMARDs), glucocorticoids and biological agents. However, the results of this therapy are unsatisfactory because few drugs are remittive and tolerable simultaneously in influencing the joint destruction and declining functional state (Lee and Weinblatt, 2001). Anti-inflammatory and analgesic drugs usually have obvious side effects and systematic complications due to their non-specific distribution. For example, utilizing drug through the oral and parenteral route cannot achieve the expected therapeutic effects because of the first-pass effect, low bioavailability, rapid metabolism, poor absorption, and serious adverse effects caused by non-specific distribution (Qindeel et al., 2020b). Surgical treatment often causes great trauma and extensive adhesion after operation. Also, surgery cannot be performed repeatedly (Yasutaka et al., 2016). Compared with traditional surgery, arthroscopic synovectomy truly has some advantages, however, it still have some blind spots (Vivek et al., 2016). Other treatment measures such as chemical synovectomy and radio-surgical resection can reduce joint exudation and prevent cartilage protection, but they could cause damage to other normal tissues (Winkler et al., 2013). In recent years, researchers focused on several other ways of giving the drug like the transdermal drug delivery system (TDDS), which can improve the permeation of therapeutic drugs through skin with higher specific distribution so the patient compliance and comprehensive therapeutic benefit will be enhanced (Prausnitz and Langer, 2008). However, it is difficult to transport macromolecules at present and its safety should be fully evaluated from the aspect of formulation. Overall, it is of the utmost importance to find an improved therapy with wonderful curative effect and less adverse reactions.

Liposomes are microsopic phospholipid bubbles whose core is encapsulated in a lipid bilayer. They can be applied as the drug carriers due to this special structure. Also, liposomes can be absorbed by the reticuloendothelial system (RES) cells in synovial tissue of rheumatoid arthritis patients (Xun and Yang, 2018). However, nonspecific uptake of RES in the liver or spleen rapidly eliminate the liposomes from the circulation. Recently, nanoparticles also show great potential in the delivery of drugs. But the poor drug loading capacity and the stability issues during storage limit their applications, such as drug release and particle gelation. Polymeric micelles have some blind spot despite their suitable structure and size (Torchilin, 2007). Hydrogels, the special type of hydrophilic polymers, are unique crosslinked three-dimensional networks that have great material exchange capacity, biocompatibility, biodegradability, and adjustable mechanical properties. However, hydrogels also have some limitations. Most hydrogels lack the ability of self-repairing,which may cause mechanical damage during implantation prosess or bone activity. Also, the high cost and complex preparation process hinder the further application.

They are generally classified into natural, semi-synthetic and synthetic polymers according to their source, which have a promising application prospects in tissue engineering regeneration and repair (Peppas et al., 2006). Researchers have modified various hydrogels to improve their comprehensive properties for clinical applications. For example, Short chain chitosan (CS) is incorporated into a covalent tetra-armed poly (ethylene glycol) (tetra-PEG) hydrogel to achieve the required mechanical properties and biocompatibility simultaneously (Yunfan et al., 2021). Also, the hydrogels based on PEG show wonderful performance in safety, injectability, good mechanical properties and tissue adhesion (Tengjiao et al., 2021). In addition, adding natural substances with different characteristics into hydrogels can comprehensively improve their performance, such as hyaluronic acid (HA) and chondroitin sulfate (Gerke et al., 2022). Xu et al. combined albumin and methacryloyl transparent hydrogels to achieve the comprehensive properties (Mengdie et al., 2022).

With the development of artificial intelligence, many smart hydrogels are also widely studied. These smart hydrogels can respond to tiny physical and chemical stimulus in the environment, such as pH value, temperature and electric field and so on (Xing et al., 2020). Yang et al. designed a new double network (DN) hydrogel through incorporating the *in situ* formed chitosan ionic network, which showed predominant mechanical properties (Yanyu et al., 2018). Hydrogels that can respond to various stimuli are also gradually increasing, such as pressure, temperature (Shuai et al., 2022) and pH (Dou et al., 2020) and so on. Therefore, the hydrogels with different properties have broad prospects in the treatment of rheumatoid arthritis. Nowadays, there are many hydrogel products applied in the treatment of rheumatoid arthritis. Also, there is a growing trend on the concerned studies. However, the special physiological environment of rheumatoid arthritis poses a challenge to the treatment with hydrogels. Persistent synovitis is characterized by cellular hyperplasia marked angiogenesis and other complex conditions, which complicate the required comprehensive properties of the hydrogel products. Natural hydrogel has good biocompatibility and safety. However, its mechanical properties and wear resistance are far from those of normal cartilage. Therefore, it must be modified for clinical applications. Therefore, it is of vital significance to develop the composite hydrogels with excellent comprehensive properties for the different treatment mechanisms of rheumatoid arthritis.

In this study, we reviewed the pathological changes of rheumatoid arthritis and the mechanisms that affect the treatment of hydrogels (Table 1, Supplementary Material). Also, we summarized the properties of hydrogels needed for the treatment of rheumatoid arthritis. Finally, we put emphasize on the introduction of hydrogels with different properties that are expected to be used in the treatment of rheumatoid arthritis through various mechanisms, thus satisfying the requirements of clinical treatment in rheumatoid arthritis.

**TABLE 1:** The outline table of hydrogels applied in the treatment of rheumatoid arthritis.

Style of delivery	Drugs or cells		Design /manufacturing methods	Outstanding properities	References
Injectable hydrogels	DMARDs	Methotrexate	Combining black phosphorus nanosheets and platelet-rich plasma-chitosan thermoresponsive hydrogel	Excellent ability of drug releasing.Thermoresponsive	Pan et al. (2020)
Injectable hydrogels	DMARDs	Methotrexate	In situ gelation and employ Au NPs	Improved cytocompatibility,modulus and cell adhesion;Excellent ability of drug releasing	Nutan et al. (2020)
Injectable hydrogels	DMARDs	Methotrexate	G^D^F^D^F^D^Y were used to create a supramolecular self-assembling hydrogel	The excellent ability to control the proliferation and migration of rheumatoid arthritis synoviocytes	Ma et al. (2022)
Injectable hydrogels	DMARDs	Methotrexate	A novel click-crosslinked HA (Cx-HA) depot was developed through the click crosslinking reaction between tetrazine-modified HA and trans-cyclooctene-modified HA	Excellent ability of drug releasing	Seo et al. (2019)
Injectable hydrogels	DMARDs	Methotrexate	Oligochitosan and hypromellose phthalate-based polyelectrolyte complexes	Thermosensitive.Excellent ability of drug releasing.	Agostini et al. (2021)
Injectable hydrogels	DMARDs	Methotrexate	Methotrexate and transforming growth factor β1 are loaded in nano-Fe_3_O_4_ composite chitosan-polyolefin to construct a multifunctional hydrogel	Inhibit inflammation and promote cartilage regeneration.Excellent mechanical properties	Gang et al. (2021)
Injectable hydrogels	DMARDs	Methotrexate	The MTX and HA loaded drug-encapsulating NPs based on PLGA and DOTA were combined to synthesize the 177Lu-DOTA-HA-PLGA (MTX)	Bi-modal mechanisms of treatment through Lutetium177 and MTX.	Trujillo-Nolasco et al. (2019)
Transdermal hydrogels	DMARDs	Methotrexate	Creating the PCL- PEG-PCL triblock copolymer through the reaction of ring opening copolymerization and applied it in the delivery of the fabrication loaded with MTX nanomicelles	Nanomicelles loaded;excellent non-newtonian property;the ability to overcome the skin barrier and relese the drug in a sustain way	Qindeel et al. (2020a)
Transdermal hydrogels	DMARDs	Methotrexate	Combining a patch-like reservoir loaded with MTX (MTX-RV) and the hydrogel-forming microneedle arrays (HFMN)	The ability to overcome the skin barrier and relese the drug in a sustain way.Negligible side effects on skin	Tekko et al. (2020)
Injectable hydrogels	DMARDs	Iguratimod	Encapsulating IGUR in biodegradable polyvinyl alcohol micelles and load it into the in situ injected hyaluronic acid hydrogels	Excellent mechanical properties and the ability of drug releasing	Ma et al. (2019)
Injectable hydrogels	NSAIDs	Ketoprofen	Transforming the anti-inflammatory drug ketoprofen into the self-assembled products through covalent conversion	Excellent ability of drug loading and releasing	Chen et al. (2017)
Transdermal hydrogels	NSAIDs	Ibuprofen	The Eudragit® L 100 (EL 100) NPs containing IB were loaded into Carbopol? 934-based hydrogel	PH-responsive.The ability to overcome the skin barrier and relese the drug in a sustain way	Khan et al. (2021)
Transdermal hydrogels	NSAIDs	Aceclofenac	Nanostructured lipid carrier (NLC)-based ACE (ACE-NLC) hydrogel	The ability to overcome the skin barrier and relese the drug in a sustain way.Nanoscale lipid carrier	Garg et al. (2021)
Injectable hydrogels	Glucocorticoids	Glucocorticoids	Combining the thermo-responsive hydrogels with microparticles	Pseudoplastic behaviors.Good biological adhesion.The ability of sustain drug releasing	Abou-EiNour et al. (2020)
Injectable hydrogels	Anti-TNF-α drugs	Anti-TNF-α drugs	A promising drug system based on anti-TNF-α chondroitin sulfate (CS) modified poly(amidoamine) (CS/PAMAM) dendrimer NPs	Good biocompatibility.Nanoscale lipid carrier	Oliveira et al. (2021)
Injectable hydrogels	Anti-TNF-α drugs	Infliximab	Incorporating the infliximab into a thermoresponsive hydrogel synthetized by combining the pluronic F127 and HA with poly (γ-glutamic acid) (PGA)	Excellent biodegradability, biocompatibility, and the ability to continuously release	Chen et al. (2020)
Injectable hydrogels	Chinese medicine preparation	Sanwujiaowan	Constructing the deep eutectic solvent (DEC) extract complex by heating the herb extracts with amino acid and citric acid.Then the DES-extract complex was introduced into the suitable hydrogel	Improved viscoelastic and mechanical properties	Xiao et al. (2022)
Injectable hydrogels	Chinese medicine preparation	Triptolide	The HA hydrogel-loaded RGD-attached AuNPs containing the TP	Nanoscale lipid carrier.Delivering heat and drugs simultaneously	Li et al. (2022)
Injectable hydrogels	Chinese medicine preparation	2-chloro-N(6)-cyclopentyl adenosine	Combining the nanocomposite hydrogel and acupuncture to improve the applications of TP in rheumatoid arthritis	The hydrogel was administrated at ST36 with a controllable and sustained release of drugs	Ren et al. (2021)
Injectable hydrogels	Multiple drugs	Indomethacin and methotrexate	A temperature-sensitive hydrogel (D-NGel) containing nanoparticles (D-NPs), simultaneously delivering IND and MTX	Temperature-sensitive.Co-delivery of indomethacin and methotrexate	Yin et al. (2020)
Injectable hydrogels	Cells	ADMSCs	A kind of hydrogel with the ability of self-healing through the dynamic reactions between infliximab and modified polysaccharides	self-healable, anti-inflammatory, biocompatible, and biodegradable properties	Zhao et al. (2021)

## 2 Properties of hydrogel materials required for treatment of rheumatoid arthritis

Rheumatoid arthritis is mainly characterized by persistent synovitis. Histologically, it exhibits cellular hyperplasia, angiogenesis, an influx of inflammatory leukocytes, and changes in the expression of cell-surface adhesion molecules, proteinases, many cytokines, and proteinase inhibitors (Lee and Weinblatt, 2001). At the same time, synovial changes in rheumatoid arthritis vary with the progression of disease. The particular special structure and inflammatory environment pose a challenge for the drug and cell therapies of rheumatoid arthritis. Therefore, hydrogels for rheumatoid arthritis treatment should have excellent comprehensive properties to meet these challenges.

We summarized some properties of hydrogels for the treatment of rheumatoid arthritis. Firstly, good biocompatibility is the basic and most important property of every kind of hydrogel. Secondly, hydrogels must have good mechanical properties, mainly including Young’s modulus, rheological properties (Jiaqi et al., 2021), fatigue properties (Diego et al., 2021), toughness and swelling ratio and so on. Thirdly, tribology properties and wear resistance are also important properties of hydrogels applied in the treatment of rheumatoid arthritis. Fourthly, biodegradability and the ability to load and release drugs or cells count. Lastly, the ability to overcome the skin barrier is of vital importance to the transdermal hydrogels.

In the current studies, there are mainly two types of hydrogels for the treatment of rheumatoid arthritis. One is intra-articular injection hydrogel, and the other is transdermal hydrogel. These two kinds of hydrogels have their own characteristics. Injectable hydrogels have shown wonderful application prospects in the controlled delivery of therapeutic agents and cell, tissue culture, and wound healing process (Bhingaradiya et al., 2020). In order to be better applied in the treatment of rheumatoid arthritis, injectable hydrogel system should have the following characteristics. The first requirement of injectable hydrogel systems is that their precursor solution should have low viscosity for injection and generate little heat during the hydrogelization. Secondly, it should be gel rapidly under the physiological conditions to reduce the release of encapsulated drugs or cells and the diffusion of solutions (Boaz et al., 2013). Thirdly, this hydrogel must be degraded in an appropriate way to promote the sustained release of drugs or nutrients. Last but not the least, hydrogels and the species produced by their degradation must be cytocompatible. In practical applications, the properties of the injectable hydrogels should be manipulated according to different types of applications. Injectable hydrogels should, for example, have higher mechanical properties than culturing tissues (Wissam et al., 2019). For the application of delivering drug, the prepolymer should be capable of loading therapeutic drugs, including hydrophilic and hydrophobic drugs. Additionally, it should also be possible for the hydrogel to release the molecules for a sustained period of time.

In addition to injectable hydrogels, many transdermal hydrogels have recently been studied and applied for the treatment of rheumatoid arthritis. Transdermal administration can avoid nonlinear pharmacokinetics and the gastrointestinal tract, so as to minimize the gastrointestinal side effects. At the same time, transdermal administration has an extended duration of action and can also avoid the pain of subcutaneous injection, reducing the risk of related complications and improving the patient compliance (Rachna and Veena, 2012). However, the strong barrier of skin and physicochemical properties of therapeutic drugs hinder the wide application of transdermal delivery systems. In addition, the transport of macromolecules is limited in transdermal administration. Therefore, the transdermal hydrogels for the treatment of rheumatoid arthritis should have the following biological properties. First of all, transdermal hydrogels must be biocompatible, and they should have negligible irritation and toxicity to the skin. Secondly, hydrogels must improve their permeability to break through the skin barrier and enter the joints to play a role. Finally, they should have a high drug encapsulation rate to maintain the required concentration for a sustained period.

## 3 Treatment strategy of hydrogel in rheumatoid arthritis

### 3.1 Improved hydrogels for the delivery of disease modifying antirheumatic drugs

Rheumatoid arthritis is primarily treated with DMARDs. They remain the first-line agents in clinics despite being poorly understood in terms of their mechanisms of action. DMARDs are one kind of drugs which could delay the progression of rheumatoid arthritis, improve the function of joint and limit progressive joint damage through reducing joint swelling and pain and decrease acute-phase markers (David et al., 2010).

The most commonly used DMARD is methotrexate (MTX). There are several studies showing that MTX consistently improves the functional status by 50–80% compared to baseline (Tugwell et al., 2000). In clinical practice, over 10 mg of MTX a week is generally required in clinical treatment and to achieve maximum effects, many patients need to increase the dosage to 15–25 mg per week (David et al., 2010). The onset of action usually takes four to 8 weeks and the dose of MTX should gradually increase until the maximum dose is reached or the significant benefit is achieved. There are several factors that hamper its clinical application through oral administration, including poor solubility, short plasma half-life, less permeability, and reduced bioavailability. MTX, on the other hand, is rapidly cleared from the body when it is injected intraarticularly. Therefore, almost 30% of patients must give up MTX therapy, meanwhile 50% of patients require high-dose MTX, increasing the risk of toxicity associated with the drug dose (Bhakti et al., 2019).

#### 3.1.1 Injectable hydrogels for methotrexate delivery

Injectable hydrogels play a role mainly through injecting into joints and gel *in situ*. Pan et al. (Wenzhen et al., 2020) developed a new thermoresponsive hydrogel that combined platelet-rich plasma (PRP)-chitosan and black phosphorus nanosheets (BPNs), exhibiting the promising results in the treatment of rheumatoid arthritis. This hydrogel can not only play a role in anti-inflammatory, but also promote intra-articular osteogenesis. In this hydrogel, BPNs can generate local heat under the infrared irradiation and deliver reactive oxygen species (ROS) to the inflamed joints, which can remove the hyperplastic synovial tissue from these joints. Besides, chitosan hydrogels could be effectively enhanced by PRP for mesenchymal stem cell adhesion and capacity. The experimental results in mice showed that the joint surface injected with this hydrogel was smooth, showing good lubrication effect and reducing joint surface damage. This smart hydrogel has excellent ability of drug releasing and is thermoresponsive and can promote the adhesion of mesenchymal stem cells. Therefore, the BPNs/Chitosan/PRP thermoresponsive hydrogels exhibited great potential in the treatment of rheumatoid arthritis through anti-inflammatory and osteogenesis mechanisms.

**FIGURE 1:**
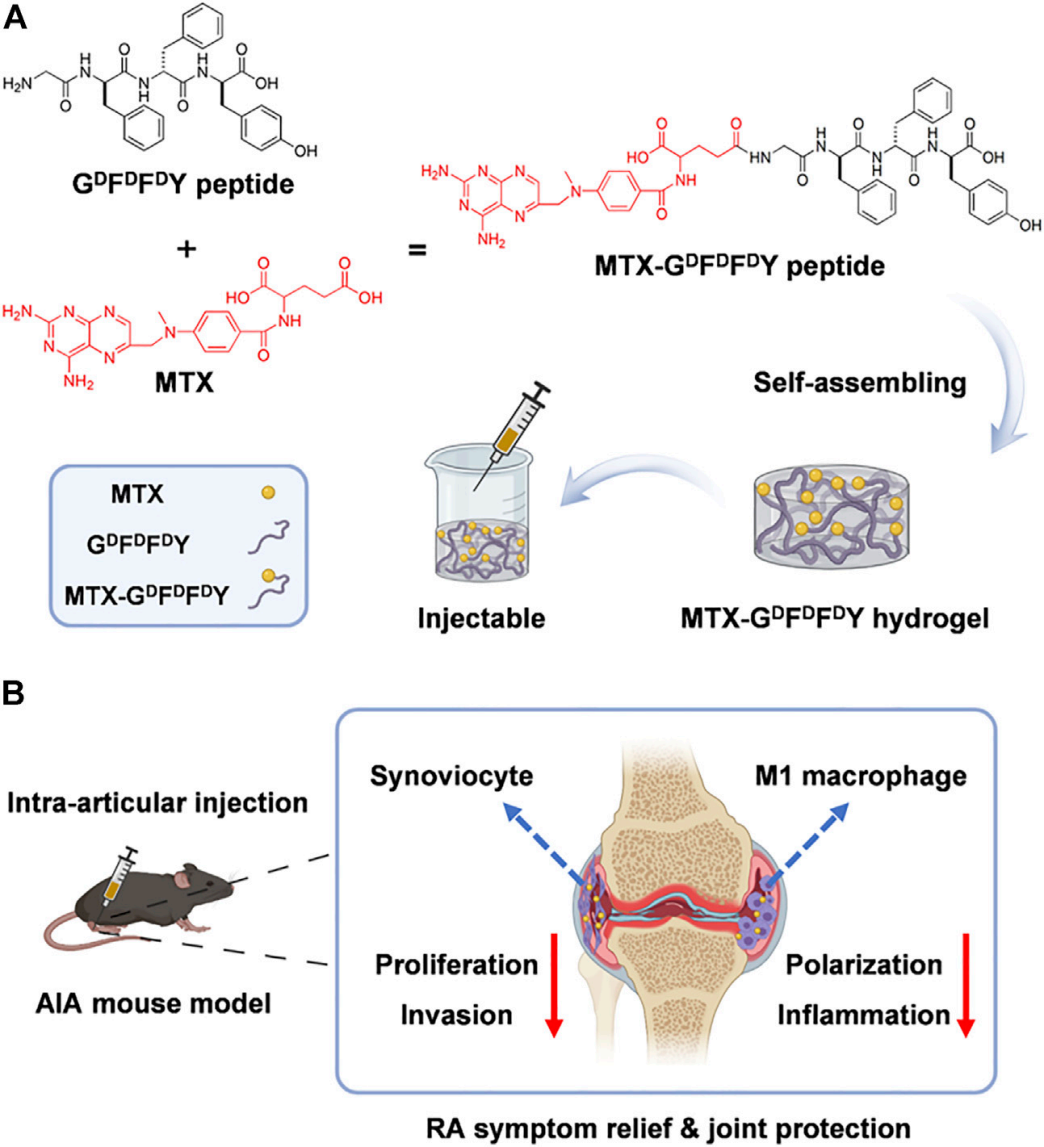
The synthesis of MTX-G^D^F^D^F^D^Y hydrogels and their use in the treatment of rheumatoid arthritis (RA) are shown schematically. **(A)** The interaction of the carboxyl groups in MTX with the deaminated glycine in peptides results in the conjugation of MTX with G^D^F^D^F^D^Y peptides. And the peptides could form injectable hydrogels through the stacking of π-π. **(B)**After being intra-articularly injected into the knee joints of adjuvant-induced arthritis (AIA) mice, the hydrogels can constantly release MTX during biodegration, and thus inhibit not only the poliferation and invasion of synoviacytes, but also the polarization of poimflammtory M1 type macrophages, achieving a highly efficent rheumatoid arthritis therapy by reducing joint inflammtion and destruction. Reproduced with permission from Ma et al., Materials Today Bio; published by Elsevier, 2022.

The gelation of hydrogels in biological environments must be accelerated and their comprehensive biological properties must be improved. Nutan et al. (Bhingaradiya et al., 2020) reported a novel gold (Au) nanocomposite hydrogel loaded with MTX, which could be gelling rapidly *in vivo* with the increased modulus, cytocompatibility, and cell adhesion when compared with pristine hydrogels. The improvement of these properties is attributed to the application of Au nanoparticles (NPs) in preparing hydrogels. Additionally, AuNPs showed the positive outcomes in treating rheumatoid arthritis (Hwiwon et al., 2014). The results of rat experiments also demonstrated that co-release of AuNPs and MTX from the kind of nanocomposite hydrogel could maintain for an extended period. The modulus, cell adhesion, cell proliferation, and cytocompatibility were greatly improved through the addition of Au NPs.This work combined gold NPs and injectable hydrogel for the treatment of rheumatoid arthritis, providing a new way for improving the comprehensive performance of drug-delivered hydrogels. D-amino acids having the sequence G^D^F^D^F^D^Y were used by (Shaodan et al., 2022) to create a supramolecular self-assembling hydrogel (Figure 1). Then, MTX was loaded into the hydrogel composite. The experimental results showed that hydrogels exhibited excellent drug selectivity, increasing MTX toxicity to rheumatoid arthritis synoviocytes while decreasing its toxicity to other normal cells. In addition, MTX-G^D^F^D^F^D^Y hydrogels showed good performance in reducing joint inflammation via effectively controlling the proliferation and migration of rheumatoid arthritis synoviocytes as well as proinflammatory M1 macrophage polarization. In the experiments of adjuvant induced arthritis (AIA) mice, this hydrogel significantly relieved joint swelling and fever. Therefore, the MTX-G^D^F^D^F^D^Y hydrogels show promising application prospects as a novel drug delivery system in the treatment of rheumatoid arthritis.

Hyaluronic acid (HA) has been considered as one of the most suitable biomaterials with comprehensive properties for all kinds of biomedical applications, which has the ability to alleviate cellular inflammatory reaction and heal the diseased tissue, and the good biocompatibility (low toxicity and immunogenicity). However, intra-articular injected HA disappears quickly at the site of injection due to the endogenous degradation of the hyaluronidase (Guidolin and Franceschi, 2014). Therefore, modifying HA hydrogels to increase the *in-vivo* residence time is of great importance. Seo et al. (Jiyoung et al., 2019a) developed a novel click-crosslinked HA (Cx-HA) depot through the click crosslinking reaction between tetrazine-modified HA and trans-cyclooctene-modified HA for the treatment of rheumatoid arthritis. The Cx-HA depot showed greatly improved properties and a longer retention time *in vivo* than common HA depot. When the hydrogel was loaded with MTX, the injectable hydrogel could rapidly form a MTX-Cx-HA depot in the joint and last for long time at the injection site. The *in vivo* result of MTX biodistribution shows that it can maintain a relatively high concentration of MTX at the intra-articular injection site for a period of time, with little MTX distributing to surrounding normal tissues and organs. Therefore, this hydrogel could maintain the therapeutic MTX concentrations for a period of time to induce the repair of joints and its adverse effects on other tissues are almost negligible. And it is characterized by its specific distribution and excellent biodegradability.

Agostini et al. (Sandra et al., 2021) proposed three MTX loaded drug delivery systems (DDS) for the treatment of rheumatoid arthritis, including: hypromellose phthalate-based polyelectrolyte complexes (MTX-PEC) and a poloxamer based thermosensitive hydrogel (MTX-HG), oligochitosan and the association of them (MTX-PEC-HG). Then, they compared the comprehensive properties of the three different delivery systems through a series of experiments. The plasmatic IL-1β level reflects the performance of MTX delivery systems and checking the tibiofemoral joint to evaluate their effects. Results showed that MTX-PEC was the best DDS to decrease pain and repair cartilage damage, with improved targeted drug release and decreased adverse reactions, which attributed to the excellent ability of drug loading and releasing and the specific distribution. These advantages over systemic oral administration may attribute to its direct effect on articular cartilage and reduced systemic exposure. Additionally, the electrostatic interaction between the positively charged nanocarriers and the negatively charged extracellular matrix is also conducive to the transmission of neutral molecules (Ambika and Alan, 2017). Thus, this hydrogel provides a promising thought for the production of hydrogels.

Gang et al. (2021) developed the multifunctional drug-loaded hydrogel (DN-Fe-MTX-TGFβ1), which combined the tissue engineering and thermochemotherapy to deal with rheumatoid arthritis effectively. This drug loaded hydrogel integrates magnetic nano-Fe_3_O_4_ and CS-polyolefin into a DN hydrogel with excellent mechanical properties, and it was loaded with transforming growth factor β1 (TGF-β1) and MTX. Hence the hydrogel can serve as an excellent carrier to continuously release TGF-β1 and MTX to supply sustained local thermochemotherapy, improve inflammatory symptoms, and slow the progression of joint erosions. In addition, some *in vivo* experiments indicated that the composite hydrogel can synergistically inhibit inflammation and promote regeneration of articular cartilage. Also, this hydrogel shows excellent mechanical properties and the ability to load and release drugs. The results show this kind of composite hydrogel combining thermo-chemotherapy and tissue engineering provides an insight on the therapeutic hydrogel with multiple mechanisms.

Lutetium177 (177Lu), a theragnostic radionuclide, could launch β radionuclides to reduce the inflammation of chemical membrane tissue when injected into intraarticular. Therefore, the targeted transport of radiotherapy agents and DMARDs to the expected area in the joint can achieve the synergistic effects of two different mechanisms. Trujillo-Nolasco et al. (Maydelid et al., 2019) combined the MTX and HA loaded drug-encapsulating NPs based on poly lactic-co-glycolic acid (PLGA) polymer and 1,4,7,10-tetraazacyclododecane-1,4,7,10-tetraacetic acid (DOTA), a complex agent of 177Lu, to synthesize the 177Lu-DOTA-HA-PLGA (MTX). This composite is characterized by specific target recognition. Its functions are based on dual mechanisms employing 177Lu for the intraarticular radio-synovectomy and MTX as DMARDs to delay the progression of rheumatoid arthritis. The results of *in vitro* experiments showed that decreased cytotoxic effect and the uptake of MTX by NPs were enhanced. Thus, the novel drug delivery with bi-modal mechanisms shows a potential in the treatment of rheumatoid arthritis.

#### 3.1.2 Transdermal hydrogels for the delivery of methotrexate

In addition to the injectable hydrogels, MTX is also delivered to the target area through the transdermal way. To enhance the efficacy of MTX, Qindeel et al. (2020a) created the polycaprolactone-polyethylene glycol-polycaprolactone (PCL- PEG-PCL) triblock copolymer through the reaction of ring opening copolymerization and applied it in the delivery of the fabrication loaded with MTX nanomicelles. After optimized, the nanomicelles were loaded into the carbopol 934-based hydrogel containing eucalyptus oil (improve the ability of NPs to penetrate the skin) as a penetration enhancer. Rheumatoid arthritis mice model experiments showed that compared with other tissues and organs, the accumulation degree of nanomicelles in inflamed joints is the highest. Meanwhile, the pharmacokinetic of MTX was greatly improved by this nanomicelles (the half-life was prolonged by 4.34 times, AUC0-t was increased by 3.68 times, and the average residence time increased by 3.15 times) when compared with free MTX. Also, this hydrogel did not activate the immune system and exhibited the remarkable decreased hepatotoxicity. Also, this hydrogel showed excellent non newtonian property and the ability to overcome the skin. To sum up, the MTX-loaded nanomicelles-based hydrogel shows excellent performance in the delivery and release of MTX.

Tekko et al. (Ismaiel et al., 2020) developed a new hydrogel to sustainably deliver MTX through the way of transdermal, which combined a patch-like reservoir loaded with MTX (MTX-RV) and the hydrogel-forming microneedle arrays (HFMN). After the fully characterization, the MTX-RV and HFMN were combined to produce the integrated patch. The results of their experiments showed that a large dose of MTX (150.3 ± 5.3 μg/mg) was incorporated into the MTX-RV without precipitation and the integrated patch can deliver MTX steadily. Furthermore, the HFMN can be completely removed from the skin, exerting little effect on the human body in the form of slight erythema on skin. Therefore, the minimally invasive transdermal drug delivery system could overcome the skin barrier and constantly deliver MTX for a period of time, providing a promising idea for the transdermal hydrogels.

In general, MTX is the main drug for the clinical treatment of rheumatoid arthritis. The objective of injectable or transdermal hydrogels is to make MTX continuously released in joints to maximize the therapeutic effect and reduce the adverse effects of drugs on other organs or systems. Therefore, in addition to some necessary properties, this kind of hydrogel needs to be continuously enhanced to improve the encapsulation rate of MTX, and enable it to form a stable drug concentration in the joint to meet the needs of treatment.

#### 3.1.3 Hydrogels loaded with other disease modifying antirheumatic drugs

Iguratimod (IGUR), a novel DMARD, shows great potential in the treatment of rheumatoid arthritis via inhibiting the production of cytokine and promoting bone formation and its anti-inflammatory effect (Mucke, 2012). However, like other DMARDs drugs, traditional oral administration of IGUR will result in serious adverse effects. Therefore, it is of great importance to design new DDS to improve its application. Ma et al. (Zhenzhen et al., 2019) encapsulated IGUR in biodegradable polyvinyl alcohol micelles, after that it was loaded into the *in situ* injected hyaluronic acid hydrogels. This HA hydrogel was cross-linked by PEG (Thiol) 2 (HS-PEG-SH). Drug release experiments showed that it can release IGUR in PBS from the composite for a sustained period (up to 72h). Collagen-induced arthritis (CIA) rats were treated with the NanoIGUR-loaded hydrogel for the evaluation of its properties. The results showed that compared with oral administration, the dosage of NanoIGUR-loaded hydrogel was reduced while achieve the same efficacy. Thus, this kind of hydrogel shows a promising application prospect in the delivery of specific DMARDs because of its excellent mechanical properties and the ability of releasing drugs in a stable way.

### 3.2 Improved hydrogels for the delivery of non-steroidal anti-inflammatory drugs

During the inflammatory reaction of rheumatoid arthritis, membrane phospholipids are released into the body with the destruction of the cell membrane, and are converted into arachidonic acid under the function of enzymes. Arachidonic acid produces prostaglandins (PG), prostacyclin and thromboxane 2 under the function of cyclooxygenase (COX). Among them, PG has high biological activity, involving in many physiological and pathological processes, such as fever, pain, inflammation, thrombosis, anaphylaxis and so on. NSAIDs play an anti-inflammatory and analgesic role by interfering with arachidonic acid metabolism. Therefore, it is widely used in the treatment of rheumatoid arthritis. However, its non-specific mechanism of action often brings many side effects, such as gastrointestinal reactions, kidney damage and so on. Therefore, improving targeting is the key to improve the application of NSAIDs in rheumatoid arthritis.

Chen et al. (Zhipeng et al., 2017) transformed the anti-inflammatory drug ketoprofen into the self-assembled products through covalent conversion that improve the selectivity and potency of ketoprofen. This kind of ketoprofen hydrogelator is capable of reducing the concentration of inflammatory cytokines (such as TNF α and IL-1), inducing the apoptosis of fibroblast like synoviocytes without destroying its biocompatibility with normal chondrocytes. Additionally, after incorporating the L- or D-amino acids, ketoprofen has a stronger inhibitory effect on COX-2 than COX-1, which minimizes the adverse effects connected with the inhibition of COX-1. Also, these hydrogels containing anti-inflammatory drugs could retain these drugs in the articular cavity after being injected into the joint, releasing into the plasma with a slow and steady speed, which showed the excellent ability of drug loading and releasing. These drug-loaded hydrogels provide the design guidance for the delivery of drugs.

Ibuprofen (IB), one kind of NSAIDs, is widely applied in the treatment of rheumatoid arthritis to relieve moderate to severe pain. However, the oral administration and intravenous injection often bring many adverse effects. Therefore, designing a novel drug delivery system is of urgent importance. Skin is the largest organ and has been used as an avenue for the transdermal drug delivery systems (TDDS). Compared with oral administration and intravenous injection, TDDS has better targeting and reduced adverse reactions. Meanwhile, invasive preparations of NSAIDs are not friendly to patients. Therefore, transdermal drug delivery has become the delivery method of many NSAIDs. Khan et al. (Dildar et al., 2021a) designed a PH-responsive system to transdermally deliver IB. After improved and optimized, the Eudragit® L 100 (EL 100) NPs containing IB were loaded into Carbopol® 934-based hydrogel. On the one hand, the drug profile of ibuprofen showed that the IB-NPs loaded hydrogel could respond to pH and release IB in a sustain pattern. This transdermal system also exhibited the significantly enhanced permeability of the nanocarrier system through skin under the function of argan oil. Its safety and significant efficacy have been verified in experiments. Therefore, with the ability of PH-responsive and sustain drug release, the IB-NPs loaded hydrogel is a promising candidate for the drug delivery.

**FIGURE 2:**
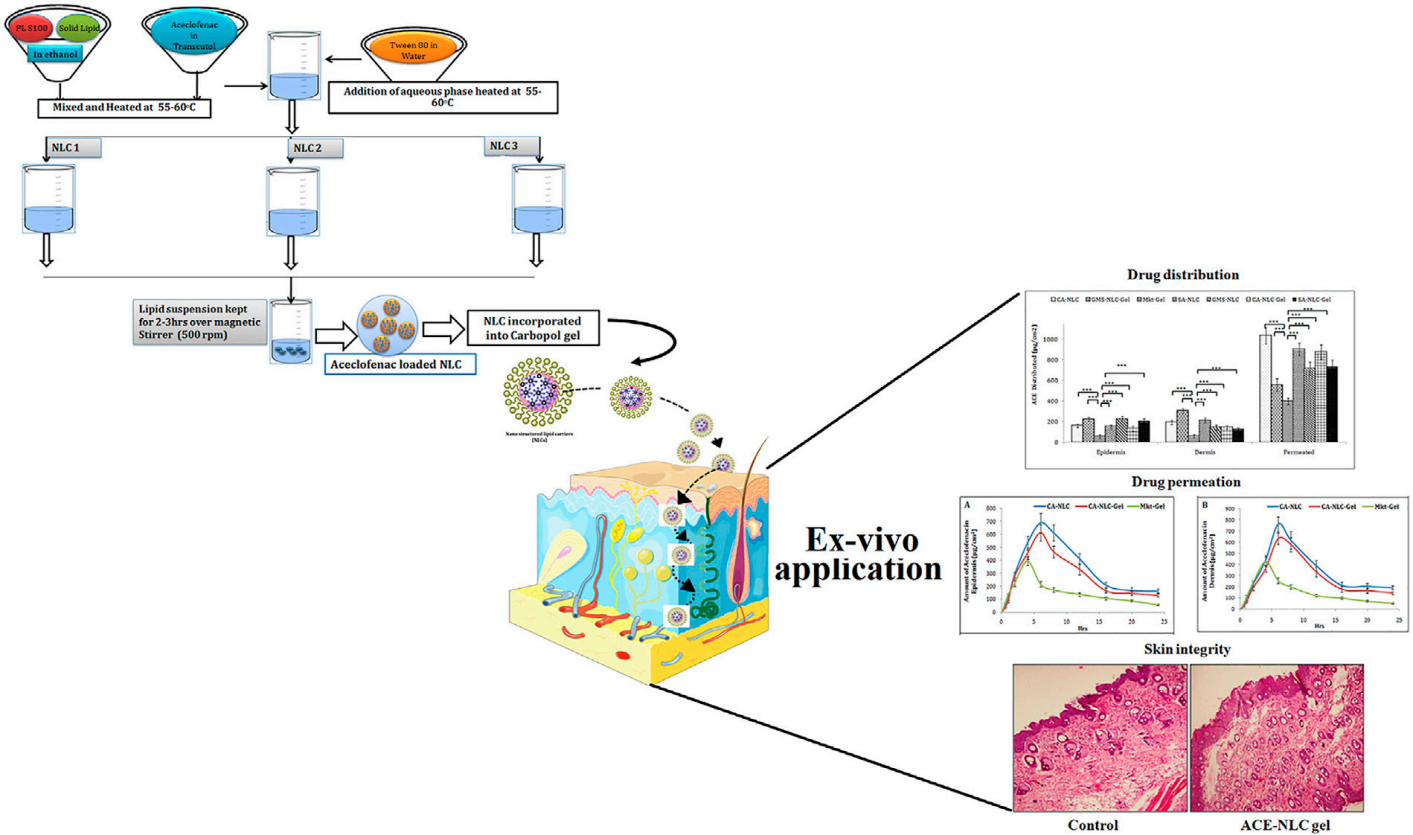
Schematic presentation of synthesis of NLCs with three different methods to produce ACE-NLC-gel and the results of transdermal applications against inflammatory disorders in animal models. Reproduced with permission from Garg et al., Original Research; published by Frontiers in Pharmacology, 2021.

Aceclofenac (ACE) is a kind of cyclooxygenase-2 inhibitor, which has been widely applied in the treatment of rheumatoid arthritis due to its effective anti-inflammatory and analgesic functions. However, traditional oral administration often causes many adverse effects, hindering the application of ACE in rheumatoid arthritis. To improve the efficiency of ACE delivery, Garg et al. (Neeraj et al., 2021) designed nanostructured lipid carrier (NLC)-based ACE (ACE-NLC) hydrogel (Figure 2). In their experiments, ACE-NLC exhibited better texture and rheological characteristics over the marketed hydrogels. Also, this hydrogel showed better skin distribution in the epidermis and dermis. *In vitro* skin permeability evaluation of ACE showed its ability to break through skin barrier through NLC-mediated ACE delivery, increasing the permeation of ACE in expected areas. In conclusion, combined with the great ability to overcome the skin barrier and relese the drug in a sustain way, this hydrogel shows a great potential to be an excellent nanoscale lipid carrier for the local application of therapeutic drugs for rheumatoid arthritis.

### 3.3 Improved hydrogel for the delivery of glucocorticoids

Glucocorticoids also play an important role in the treatment of rheumatoid arthritis. Short term glucocorticoid therapy can improve the synovitis of joints. Although the long-term use of glucocorticoids can decrease some symptoms such as joint damage, it will cause many serious side effects such as infection and osteoporosis. We usually apply glucocorticoids to treat rheumatoid arthritis in two settings. Firstly, applying glucocorticoids in the early progression of rheumatoid arthritis can improve the related symptoms rapidly. The second setting is that intra-articular glucocorticoids are used to alleviate joint related symptoms of rheumatoid arthritis (David et al., 2010). Therefore, the applications of modified hydrogels in delivering glucocorticoids to the joints show a great potential to promote the application of glucocorticoids in rheumatoid arthritis.

**FIGURE 3:**
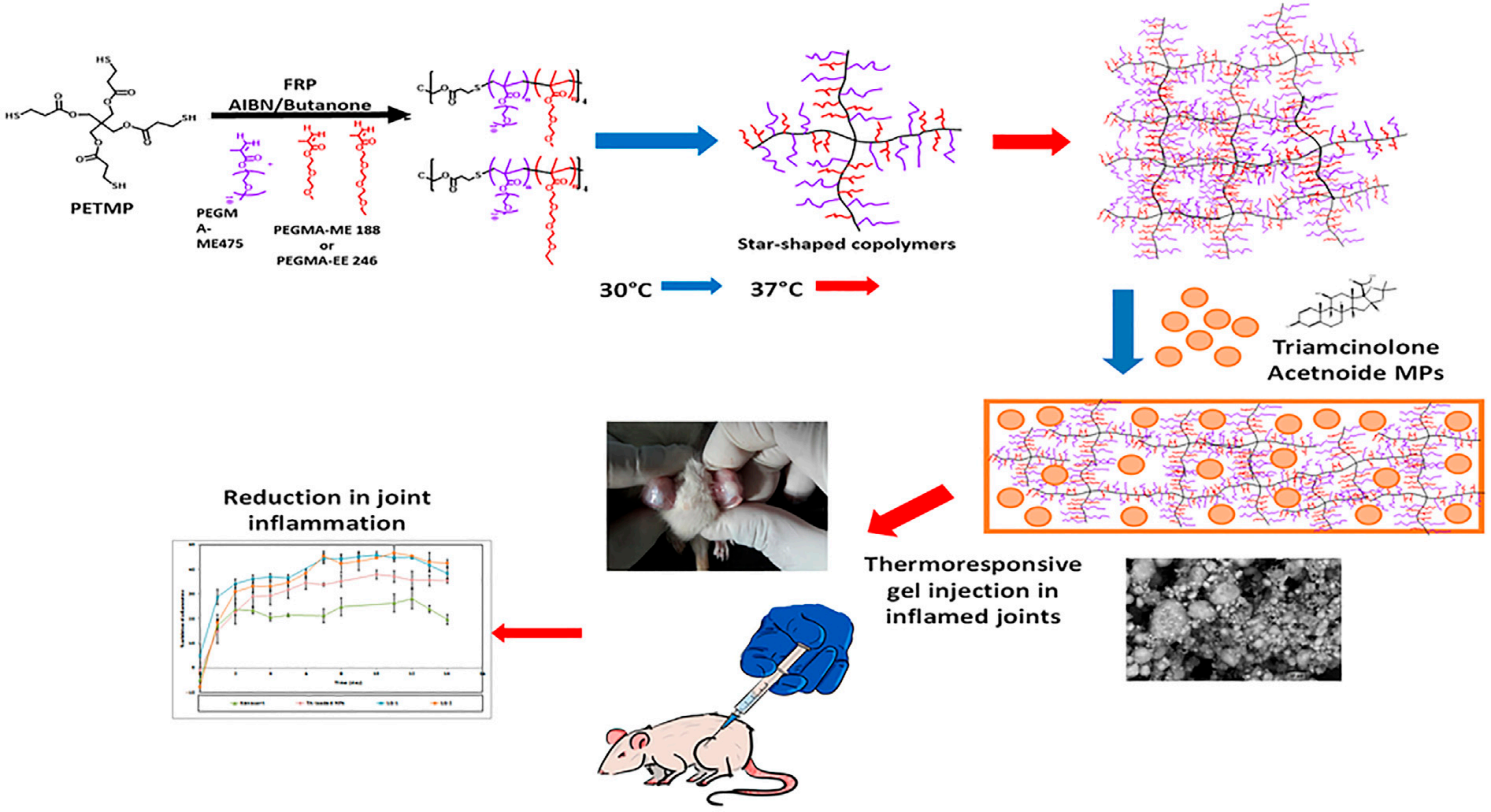
Schematic presentation of preparation of Triamcinolone acetonide (TA)-loaded MPs-in-hydrogel systems which are thermo-responsive. *In vivo* experiments showed that the reduction in joint inflammation compared with other groups after injecting this hydrogel into the inflamed joints of rats. Reproduced with permission from Abou-EiNour et al., Molecular Pharmaceutics; Published by American Chemical Society, 2020.

Abou-EiNour et al. (May et al., 2020) designed the thermo-responsive hydrogels combined with microparticles (MPs) (Figure 3), which was injected into articular to treat rheumatoid arthritis by sustaining the effect of anti-inflammatory drugs in the articular cavity. These MPs were fabricated from some polymers and the encapsulating triamcinolone acetonide (TA). The experimental results of arthritis-induced rat models showed that TA-loaded MPs-in-hydrogel systems had advantages in the alleviation of arthritis when compared with the similar product in market (Kenacort®) and MPs alone. Thus, with the great ability of sustain drug releasing, the combination of MPs and thermo-responsive hydrogels suggest a promising applicability in the application of drug intra-articular delivery.

Oliveira et al. (Isabel et al., 2021a) designed the tyramine-modified gellan gum (Ty-GG) hydrogels through the crosslinking of horseradish peroxidase (HRP), solving the problem that the injectable gellan gum (GG) hydrogels will be weaker in physiological conditions. This hydrogel showed good mechanical strength and resistance, and was capable of controlling the release of betamethasone. Betamethasone was encapsulated in the modified hydrogel to improve the specificity and safety of treatment for rheumatoid arthritis patients. The results showed that betamethasone-loaded Ty-GG hydrogels exhibited better effective therapeutic effect, without cytotoxicity and negative effects on cartilage when compared with the administration of betamethasone alone. Based on the above-mentioned advantages, the hydrogel composites have an excellent potential in the drug delivery of rheumatoid arthritis.

### 3.4 Improved hydrogel for delivery of other therapeutic drugs

#### 3.4.1 Tumor necrosis factor-α inhibitor

Tumor Necrosis Factor alpha (TNF-α), an inflammatory cytokine consisting of a trimeric protein, plays an integral role in inducing the synovitis of rheumatoid arthritis and joint destruction, resulting in joint swelling, pain and other symptoms (Mark and Baron, 2016). Therefore, many anti-TNF-α drugs have been studied and designed for the treatment of rheumatoid arthritis. Oliveira et al. (Isabel et al., 2021b) developed a promising drug system based on anti-TNF-α chondroitin sulfate (CS) modified poly (amidoamine) (CS/PAMAM) dendrimer NPs loaded into Tyramine-Gellan Gum and Tyramine-Gellan Gum/Silk Fibroin hydrogels, which improved the efficacy of treatment. This system enhanced the ability of releasing the anti-TNF-α encapsulated with NPs in a sustained and controllable manner in intra-articular sites. Also, the biological activity of antibody is maintained to immobilize TNF-α highly. This advanced approach shows great potential personalized medication with improved therapeutic results and decreased adverse effects.

Infliximab (IFX), a new monoclonal antibody drug, is the first biological-response modifiers approved for the treatment of rheumatoid arthritis. However, the long-term application of infliximab could bring many adverse effects, such as serious infections and malignancy (Yun-Chi et al., 2019). Therefore, Chen et al. (Weiying et al., 2020) incorporated the infliximab into a thermoresponsive hydrogel synthetized by combining the pluronic F127 and HA with poly (γ-glutamic acid) (PGA). This injectable hydrogel composite showed excellent biodegradability, biocompatibility, and the ability to continuously release. In the rheumatoid arthritis rabbit model, this hydrogel composite formed a local sustained-release system after intra-articular injection and significantly inhibited the expression of TNF-α in cartilage and synovial fluid, thereby inhibiting the progression of inflammation, delaying the destruction of cartilage and relieving the pain in rheumatoid arthritis. Therefore, the IFX-loaded hydrogel showed a promising future in the treatment of rheumatoid arthritis.

#### 3.4.2 Traditional Chinese medicine preparation

“Sanwujiaowan”, an important Chinese herb formula, shows potential in the treatment of rheumatoid arthritis. However, most of the raw materials used to make “Sanwujiaowan” contain strong toxicity, hindering the wide application in clinical practice. Xiao et al. (Suyun et al., 2022) constructed the deep eutectic solvent (DEC) extract complex by heating the herb extracts with amino acid (served as the receptor of hydrogel) and citric acid (served as the donor of hydrogel). Then, they introduced DES-extract complex into the suitable hydrogel, which showed improved viscoelastic and mechanical properties. In the experiments of collagen-induced arthritis rat models, this hydrogel showed enhanced therapeutic results, significantly reducing the toxicity and the inflammatory reaction of the extracts. Thus, the novel DES-hydrogel system could greatly promote the application of Chinese herb medicine in rheumatoid arthritis.

**FIGURE 4:**
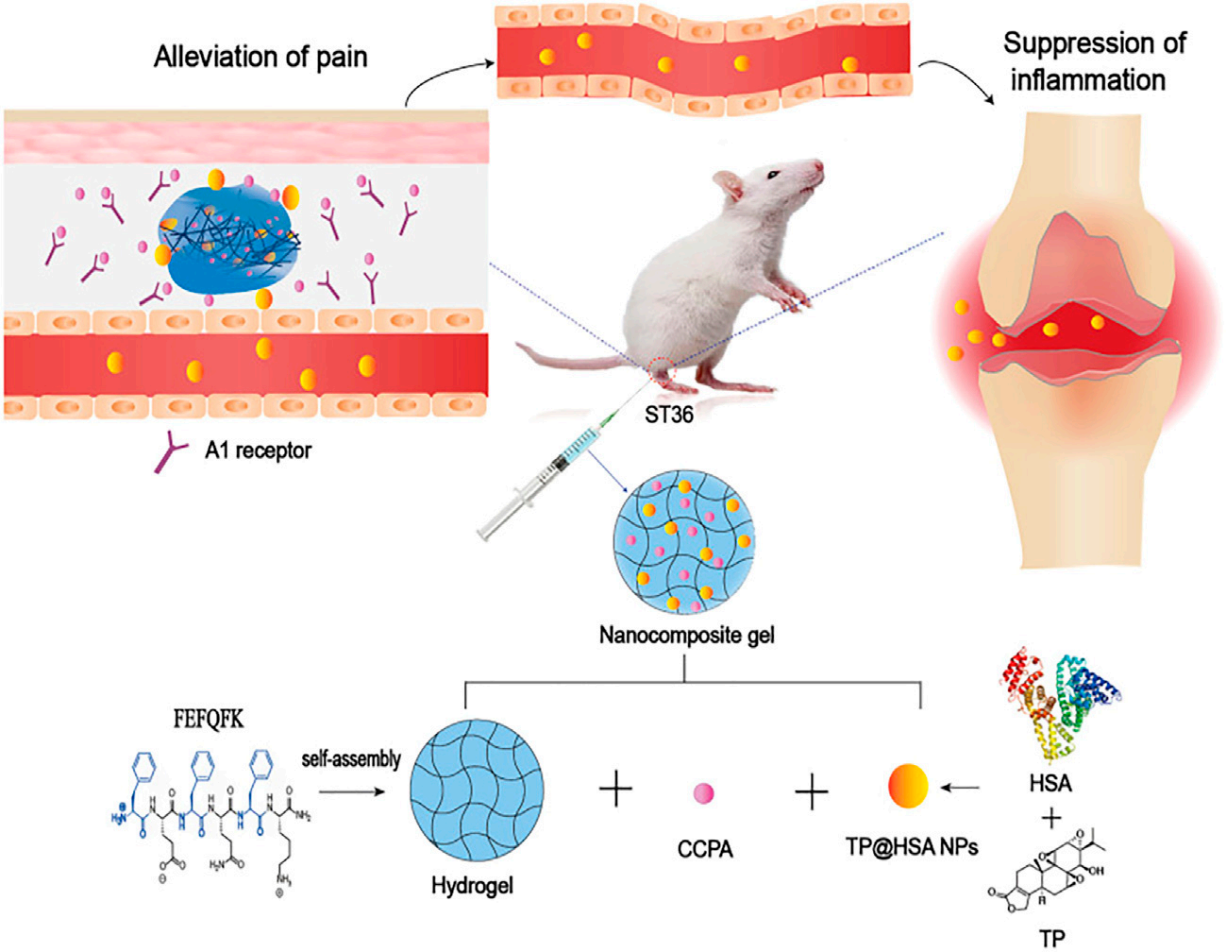
Schematic presentation of the mechanism of nanocomposite hydrogel applied in the treatment of rheumatoid arthritis. CCPA and TP@HSA NPs formed the acupoint nanocomposite hydrogel, which can simulate acupuncture to relieve pain through the stimulation of A1R, and realize the targeted deliver TP to suppress inflammation by regulating the expression level of inflammatory factors and maintaining the immune balance. Reproduced with permission from Ren et al. Journal of Nanobiotechnology; Published by BioMed Central, 2021.

Triptolide (TP), a kind of DMARDs extracted from traditional Chinese Medicine, plays a significant role in anti-inflammatory and cartilage protection via inhibiting the release of pro-inflammatory cytokines and the expression of adhesion molecules, metalloproteinases. However, the poor water solubility and severe systemic toxicity hinder its clinical applications (Zhao-Li et al., 2012). Hence, to enhance the therapeutic specificity and reduce the toxicity, Li et al. (Chenxi et al., 2022) developed a HA hydrogel-loaded RGD-attached AuNPs containing the TP. The targeted photothermal-chemo and *in-vivo* imaging of inflamed can be achieved through this hydrogel. After the intra-articular injection of hydrogel, the HA chain degrades. Under the near-infrared resonance (NIR) irradiation of AuNPs, the inflammatory area generates heat locally; the NPs could release TP and deliver heat and drugs to the joints at the same time. The results of CIA mice also showed improved therapeutic results. Therefore, the targeted photothermal-chemo therapy with improved efficacy and decreased side effects related to dosage provides a potential useful system for the targeted therapy of rheumatoid arthritis. Ren et al. (Shujing et al., 2021) combined the nanocomposite hydrogel and acupuncture to improve the applications of TP in rheumatoid arthritis (Figure 4). The 2-chloro-N (6)-cyclopentyl adenosine (CCPA) is a kind of selective A1 receptor agonist. When local administration is applied in the Zusanli point (ST36), CCPA could function as an analgesic. They used the hydrogel depot loaded with CCPA and TP@HSA (human serum album) nanocomposites to prepare the nanocomposite hydrogel (TP@HSA NPs-CCPAGel). Then the hydrogel was administrated at ST36 to get the controllable and sustained release of drugs included. The analgesia experiment indicated that the acupoint administration of this hydrogel could maintain the analgesic effect for an extended time, effectively improves joint swelling, and significantly reduces the toxic and adverse effects of TP. This new drug delivery system combined with traditional Chinese medicine deserves attention. Pham et al. (Duy et al., 2022) developed a silk fibroin *in-situ* hydrogel served as the drug delivery system of the extract of sesban for the treatment of rheumatoid arthritis. Sesbania sesban L. extract is a plant with high anti-inflammatory, which could relieve symptoms of rheumatoid arthritis. The results showed the hydrogel can be injected into intraarticular in the form of liquid formulation and quickly transformed into hydrogel and retained *in situ* for an extended time. The system can continuously release the extract for more than 20 days, showing good therapeutic activity.

### 3.5 Hydrogels for co-delivery of treatment drugs

Considering the uncertainty of rheumatoid arthritis pathogenesis and the difficulty of treatment, combination therapies have been increasingly applied in recent years to address the problems of side effects and poor therapeutic outcomes associated with the use of single drugs. Many studies show that the combination of agents and cells could exert synergistic effects on the efficiency of improving gene transfection and increasing the anti-inflammatory effect of agents. At the same time, the appropriate combination of drugs with different mechanisms could show a synergistic effect and reduce the amount of agents needed to reduce possible side effects.

Indomethacin (IND), a kind of NASID, can rapidly relieve joint swelling and pain via interfering with the metabolism of arachidonic acid. However, it could not delay the progression of rheumatoid arthritis intrinsically; MTX cannot immediately relieve pain and inflammation, but it fundamentally delays the progress of rheumatoid arthritis. Combining the two drugs in the treatment could well improve the symptoms and control the progression of disease. However, the co-administration of MTX and the IND also increase the toxicity of MTX by increasing the concentration of MTX in serum (Marinella et al., 2013). Therefore, more emphasis should focus on finding a new and efficient targeted delivery system. Yin et al. (Na et al., 2020a) developed a temperature-sensitive hydrogel (D-NGel) containing nanoparticles (D-NPs), simultaneously delivering IND and MTX for the treatment of rheumatoid arthritis. The non-covalent interactions of PEI-SS and the hydrophobic small molecule drugs contribute to the formation of D-NPs, after that the NPs were loaded into a matrix which is sensitive to temperature. The hydrogel presents a fluid state at room temperature and it is rapidly transformed *in situ* into gel after the injection into articular cavity of CIA rats, releasing drugs stably for a long time (more than 72 h). In conclusion, the symptoms of joint swelling, expression of inflammatory cytokines in joint fluid and bone erosion significantly improved by the D-NGel. This new delivery system with the ability to deliver two kinds of drugs provides inspiration for the synergistic therapy. They also (Na et al., 2020b) designed a multifunctional drug delivery system of the hydrogel loaded with PEI-SS-IND-MTX-MMP-9 siRNA nanoparticles (D/siRNA-NGel), which could realize the co-delivery of MTX, IND, and MMP-9 siRNA via multiple signaling pathways for the comprehensive treatment of rheumatoid arthritis. MTX is the first-line agent applied in the clinical treatment, which can slow down the progression of bone erosion. Abnormal secretion of MMPs in rheumatoid arthritis leads to the degradation of microvascular basement membrane and cartilage ECM. MMP-9 siRNA is a siRNA that can silence the over expression of MMP-9 gene to reverse cartilage and bone destruction. In the models of CIA mouse, they also found that D/siRNA-NGel can truly decrease the expression of inflammatory cytokines and MMP-9 in several kinds of joint fluid and plasma. Hence, this synthetic hydrogel may be really useful in the co-delivery of therapeutic drugs.

### 3.6 Hydrogels with other mechanisms

#### 3.6.1 Hydrogel loaded with mesenchymal stem cells

The application of mesenchymal stem cells (MSCs) in rheumatoid arthritis mainly focused on immunomodulatory and tissue-repair properties. MSCs could prevent the happen of joint injury through the characteristics of anti-inflammatory and immunosuppressive. Meanwhile, the protective effects of MSCs against excessive osteoclast-mediated bone resorption are likely regulated through suppressing the inflammatory cytokines. However, the application of MSCs in clinical researches is restricted to patients who have severe rheumatoid arthritis refractory to standard therapies. If the MSCs could be given at the beginning stage of the treatment of rheumatoid arthritis, this therapy maybe more effective by “resetting” the immune system (Sharon et al., 2017).

\Adipose-derived mesenchymal stem cells (ADSCs) are the promising cells applied in the regenerative medicine among all kinds of pluripotent stem cells, because ADSCs could be easily obtained from healthy donors through the minimal invasive approaches. In addition, because this kind of cell transplantation is considered to be auto transplantation and without ethical problems, so they seems really promising in clinical applications (Pedro et al., 2020). However, the widespread application of ADSCs is limited by the complex pathological conditions of rheumatoid arthritis. Excessive production of protease, intense inflammation and high levels of ROS in rheumatoid arthritis joints hinders the differentiation of transplanted ADSCs and even kills these cells. Therefore, it is of great importance to find ideal biomaterials to deliver ADSCs that could work in the micro-environment of chronic inflammation in the articular cavity. Hydrogels applied in rheumatoid arthritis treatment are mainly divided into two types, one can deliver therapeutic drugs. The other hydrogels are made from therapeutic agents and they are self-assembled or chemically crosslinked into the materials for the preparation of hydrogels where the drugs themselves are chemically crosslinked or self-assembled into a hydrogel through the interactions of noncovalent supramolecular (Fan et al., 2009). Zhao et al. (Yue et al., 2021) designed a kind of hydrogel with the ability of self-healing through the dynamic reactions between infliximab and modified polysaccharides. The designed hydrogels based on infliximab were characterized by their superior properties of anti-inflammatory,self-healable and biodegradable. When they are integrated with 3D printed porous metal scaffolds (3DPMS), the ADSCs loaded into the three-dimensional scaffold still maintained improved viability and proliferative capacity, and the differentiation of osteogenic in the complicated micro-environment. The results of their experiments also showed the relevant symptoms of rheumatoid arthritis rabbit model have been improved after the composite scaffold was implanted. For example, the inflammatory cytokines were inhibited, the subchondral bone repair was improved and even the damaged cartilage could be rebuilt gradually. With the properties of self-healable, anti-inflammatory, biocompatible and biodegradable, This inorganic-organic hybrid system provides an excellent potential therapeutic option for the stem cell therapies of rheumatoid arthritis.

#### 3.6.2 Hydrogels with the ability of nitric oxide-scavenging

Nitric oxide (NO) plays an important role in human body. But excessive production of NO can lead to rheumatoid arthritis. So it is of great importance to scavenge the excessive NO for the treatment of rheumatoid arthritis (Jiwon et al., 2019). Kim et al. (Taejeong et al., 2021) designed a polymeric aggregate-embodied hybrid NO scavenging and sequential drug-releasing “click” hydrogel (M-NO) which could combine the two different mechanisms to deal with rheumatoid arthritis. The dialkynefunctionalized NO-cleavable cross-linker (DA-NOCCL, N, N-(2amino-1,4-phenylene) dipentyn-4-amide), a biocompatible and lubricating polymer, was synthesized and incorporated to realize NO-responsiveness rapidly and selectively. Therefore, M-NO hydrogel could respond to NO, which serves as a hallmark of disease severity to release connected drugs depend on the demand and for dual-stage. The experimental results show that M-NO gel effectively alleviated the symptoms, and decreased the expression levels of NO and pro-inflammatory cytokine in mice. M-NO hydrogel provided a new idea for the treatment with hydrogels.

## 4 Challenges

So far, the properties of hydrogel materials for the treatment of rheumatoid arthritis are continuously improved. The complex physiological environment in the articular cavity of rheumatoid arthritis also puts forward higher requirements for the applied hydrogels, which require a variety of properties to achieve the better treatment of rheumatoid arthritis. These properties include good biocompatibility, strong mechanical strength, good drug or cell loading capacity, controlled and sustained drug release capacity. Therefore, it is an urgent problem that needs to be solved at present to achieve better comprehensive properties of hydrogels through the fusion of different materials. Artificial intelligence can assist us in simulating the fusion of different materials and reducing the times of experiments. Finally, we may find composite hydrogel materials with enhanced comprehensive properties suitable for the treatment of rheumatoid arthritis.

The development and treatment of rheumatoid arthritis is highly complicated, bringing about significant trouble to the treatment of rheumatoid arthritis patients. This main reason is the persistent inflammatory environment in rheumatoid arthritis joints. Persistent inflammation leads to significant angiogenesis and cell proliferation, changes in the expression of cell-surface adhesion molecules, proteases, protease inhibitors and many cytokines, and continuous destruction of bone tissue until the patient loses their joint function. However, at present, most hydrogels for the treatment of rheumatoid arthritis only deliver one type of drugs, which may be limited by the properties of hydrogels, resulting in poor treatment effect compared with the application of multiple drugs. Therefore, future studies should focus on improving the comprehensive properties of drug loaded hydrogels to co-deliver therapeutic agents with different mechanisms for synergistic treatment, so as to achieve better therapeutic effect.

## 5 Conclusion and prospects

We look back on the research progress of composite hydrogels applied in rheumatoid arthritis in the recent years. They perform a marked effect mainly through delivering different therapeutic drugs into the joint. Different hydrogels have their own characteristics. Thus, we put forward some suggestions for the following research: 1) Drug loaded NPs combined with hydrogels have shown good therapeutic effects in the treatment of rheumatoid arthritis, so we can pay more attention to this kind of hydrogels in future studies. 2) Using therapeutic drugs and polymer materials to directly synthetize hydrogels is also a promising way in the treatment of rheumatoid arthritis. Additionally, some traditional Chinese medicine preparations such as Triptolide and “Sanwujiaowan” have also shown good therapeutic effects in the treatment of rheumatoid arthritis, which is worthy of in-depth studies. 3) Smart hydrogel has a good application prospect in the future. For example,thermo-sensitive hydrogels are soluble at low temperature, which is conducive to injection, and rapidly solidify at higher temperature. Therefore, thermo-sensitive hydrogels with good biocompatibility are excellent materials for intra-articular injectable hydrogels. When we decide which hydrogel to use, in addition to considering the performance of the hydrogel, we must also pay special attention to the specific situation of rheumatoid arthritis patients and decide which treatment strategy to apply according to the different progress of rheumatoid arthritis.

Drug delivery is the main approach to treat rheumatoid arthritis with hydrogels. This method uses hydrogel as the carrier of drugs or cells to suppress inflammation and protect cartilage by loading the agents into hydrogels to function at the lesion site after implantation. Besides, hydrogels can also treat rheumatoid arthritis through other mechanisms, such as removing excessive NO in joints and implanting and promoting the culture of mesenchymal stem cells and so on. This article reviews the application of composite hydrogels for the treatment of rheumatoid arthritis and these hydrogels show broad prospects for the treatment of rheumatoid arthritis.
